# Achieving Lower District Heating Network Temperatures Using Feed-Forward MPC

**DOI:** 10.3390/ma12152465

**Published:** 2019-08-02

**Authors:** Nathan Zimmerman, Konstantinos Kyprianidis, Carl-Fredrik Lindberg

**Affiliations:** 1 Department of Automation in Energy and Environment, School of Business, Society and Engineering, Mälardalen University, Box 883, 721 23 Västerås, Sweden; 2 ABB Force Measurement, Tvärleden 2, 721 36 Västerås, Sweden

**Keywords:** district heating, DHN, control, MPC, dynamic modelling, feed-forward, energy savings

## Abstract

The focus of this work is to present the feasibility of lowering the supply and return temperatures of district heating networks in order to achieve energy savings through the implementation of feed-forward model predictive control. The current level of district heating technology dictates a need for higher supply temperatures, which is not the case when considering the future outlook. In part, this can be attributed to the fact that current networks are being controlled by operator experience and outdoor temperatures. The prospects of reducing network temperatures can be evaluated by developing a dynamic model of the process which can then be used for control purposes. Two scenarios are presented in this work, to not only evaluate a controller’s performance in supplying lower network temperatures, but to also assess the boundaries of the return temperature. In Scenario 1, the historical load is used as a feed-forward signal to the controller, and in Scenario 2, a load prediction model is used as the feed-forward signal. The findings for both scenarios suggest that the new control approach can lead to a load reduction of 12.5% and 13.7% respectively for the heat being supplied to the network. With the inclusion of predictions with increased accuracy on end-user demand and feed-back, the return temperature values can be better sustained, and can lead to a decrease in supply temperatures and an increase in energy savings on the production side.

## 1. Introduction

District heating technology is a well established process for distributing localized heat production in order to meet customers’ spacial and hot water requirements. The heat provider is able to satisfy customer demands through the development and control of a heat distribution network. The centralization of heat production is commonly generated by using combined heat and power plants (CHP), or it can be the byproduct of industrial processes. The primary benefits of district heating (DH) is that it provides lower heating costs in dense urban areas and it contributes to lower environmental impacts due to the centralization of heat production [1]. The centralization of heat and power production decreases the demand for primary energy production by introducing the opportunity to utilize excess heat in the form of a district heating network (DHN) [2]. The ability to lower the supply temperature to DHN has the possibility to not only significantly reduce greenhouse gas emissions, but it can also promote the transition towards integrating more renewable energy sources and lead to a more sustainable energy system [3].

Currently, most of the installed district heating networks are categorized as third generation DHNs. The first and second generations of district heating were based on high temperature and pressure networks, and subsequently resulted in higher losses. With third generation DH, the typical supply and return temperatures are approximately 80– 110 ${}^{\circ }C$ and 40– 50 ${}^{\circ }C$ respectively [4]. The driving force of replacing high energy content fossil fuels with lower energy bio fuels no longer constrains the temperature boundaries in a DHN. At the temperature levels mentioned above it is possible to satisfy customer needs and increase the DH system’s efficiency by operating at the lower end of the referred boundaries. The progression towards lower distribution temperatures has the ability to lower network losses, and is known as fourth generation district heating (4GDH). The underlying philosophy of 4GDH is to be able to achieve annual averages of 50 ${}^{\circ }C$ in the supply line and 20 ${}^{\circ }C$ in the return [2]. However, this approach, from the network perspective, could imply that the existing infrastructure would need to be updated, which would require a financial investment from the heat provider [5]. In recent work, Nord et al. [6] present the challenges and potential of gradually decreasing network supply temperatures. Through a series of case studies they incrementally lower the supply temperature from 80 ${}^{\circ }C$ to 55 ${}^{\circ }C$, where the trajectory of lowering the temperature is achievable given the current network structure. They found that it is possible to obtain higher heat savings, by reducing the network temperature load, with a minimal increase in the annual pump energy. The gradual progression of reducing the supply temperature is important because existing buildings are currently designed for higher temperature requirements, which amount to one third of the total energy consumed worldwide [7]. The prospects of gradually decreasing the supply temperature could greatly alleviate the financial burden of grid redevelopment. Lowering distribution temperatures to the levels of 4GDH will rely on the assumption that customer substations are working properly and that buildings meet higher energy standards. Both circumstances will be difficult to overcome when cities with existing networks are considered, due to the fact that the current share of buildings requiring higher temperatures is expected to constitute the majority of the heating demand for decades to come [8]. However, according to Gadd et al. [9] it is possible to achieve a supply temperature of 70 ${}^{\circ }C$ and return temperature of 32 ${}^{\circ }C$ within the current limitations of third generation DHNs. Furthermore, there are no documented studies of DHNs showing average return temperatures below 30 ${}^{\circ }C$ [2]. These boundaries are the focus of this work within a third generation network, while the lower temperatures of 4GDH are not considered. These boundaries are however an advancement towards achieving the temperatures of 4GDH.

The alternative to high investment costs associated with updating the infrastructure of current DHNs is to introduce more efficient control. In practice, the current mode of control is based on operator knowledge and historical consumption patterns based on outdoor temperatures. From the operator’s experience they are able to inject heat into the network before it is required with no account of how the load supplied actually affects the end-user. The downside to this approach is that even though the customers’ demands are met, the return temperature in the network will be higher than it needs to be. This implies that not only is the supply temperature often higher than necessary, but that there is great potential for reducing the DH plant load by reducing the network supply temperature. With the inclusion of more accurate feed-forward and feed-back control, the return temperature can be controlled near a set-point. This suggests that the set-point can be decreased while maintaining the thermal comfort needs of the end-user. To achieve this, model predictive control (MPC) can be utilized to meet the customers’ demands and to achieve lower return temperatures in the network. This achievement is of great interest to heat providers as it has the potential to reduce the heat load on the plant side, i.e., it would be possible to reduce peak load production during colder months by lowering the network supply temperature.

## 2. State of the Art-Background

This section is intended to enlighten the reader to previous works pertaining to the scope of this paper. The topics of modelling and control are not new concepts. However, the combination of both domains, to establish a modelling and control approach, is of significant interest.

### 2.1. Modelling

In regards to modelling heat propagation in district heating networks, two approaches come to light, which are black-box and physical modelling. Black-box models operate on the premise that with a given set of input(s) you will be able to obtain the corresponding output(s). However, the basis of such an approach mandates a need for historical process data which can be trained to fit only one case. In comparison, the development of physical models incorporates the physical aspects of a real system, and can be implemented for any case with complete transparency. With a validated physical model it is also possible to evaluate the degree at which different levels of excitation affect the defined system. This information can then be utilized in black-box system identification methods to derive a linear dynamic model around an operating point. The linear model can then be used to design an MPC controller, and in turn, be applied to the physical model. This approach is used in this paper, while previous works on the topic of modelling DHNs using physical models follows below.

Through the development of dynamic DHN models, the transient behavior of heat propagating through the network is possible to observe. It also allows for the added benefit of not only enabling the investigation of different operational regimes, but also the ongoing development of operational optimization models [10]. Giraud et al. [11] developed and validated components using Modelica, where they are able to use their model to optimize network supply temperature using actual heat load profiles. A functional comparison conducted by Soons et al. [12] compares the different platforms of Dymola (Modelica), Matlab Simulink, and TRNSYS. Their comparison indicates that Modelica performs better in terms of modularity, multi-domain modelling, and realistic control behavior. The obtainable flexibility of using an object-oriented modelling approach increases the capabilities during feasibility studies. In the work by Hermansson et al. [13] a dynamic district heating library in Modelica presents the capability of automating network modelling. By using computer-aided design drawings of a DHN a framework was developed which could automate the modelling and simulation of any network. In order to investigate the prospects of future district heating technology, fourth generation district heating, Schweiger et al. [14] and Kauko et al. [15] demonstrate that Modelica is a suitable modelling language that can efficiently simulate large scale district heating and cooling systems.

### 2.2. Control

Traditional control approaches in district heating networks are a function of outdoor temperature and the corresponding load throughout the day. This is especially apparent when comparing summer and winter customer consumption patterns. During the winter, the load from the CHP is much higher because there is a significant need for space heating (SH), and therefore the outdoor temperature captures this. During the summer, the load is significantly reduced, since the requirements of the customer needs are primarily attributed to domestic hot water (DHW) consumption, and therefore the time of the day aids in capturing this profile. According to Laakkonen et al. [16] there is a low level of utilization of automation in supply temperature controls. This is because (a) the transport delay from supplier to consumer is in a continuous flux, (b) it is difficult to estimate the consumers’ consumption profiles, and (c) there is no exact definition on optimal supply temperature. Therefore, the limits of traditional control remains to be in the hands of those in the control room. From their experience, and the weather forecast, heat is loaded into the network before it is needed, which is done in order to account for long distribution delays.

An advanced control approach, such as predictive control, can be reviewed in the work of Sandou et al. [17]. They present a method for using a Sequential Quadratic Programming algorithm in a two model approach where a closed loop simulation model is used to update an open loop optimization model. However, there was no consideration on mass flow dynamics, i.e., they assumed a constant mass flow rate. Grosswindhager et al. [18] present a method that uses Fuzzy Direct Matrix Control to control the network supply temperature. The non-linearity of the DHN, caused by the continuous flux in operating flow rate, is controlled by predicting the supply temperature at nodes within the network based on the plant supply temperature and mass flow rate.

For robust control, model predictive control (MPC) utilizes feedback from the network in order to compensate for prediction errors and model mismatches [19]. In an MPC approach by Saarinen [20] the potential of reducing the DHN supply temperature is investigated in order to evaluate the electricity production at the CHP. The use of operational data is incorporated in order to derive the heat load, return temperature, and transport delay prediction models. The control strategy proved to be favorable by increasing the potential for electricity production from a resulting lower supply temperature. In an approach where fluctuating loads are compensated for, Verrilli et al. [21] present an MPC approach, where the objective function is the associated costs for heat production and the constraints are supply temperature, generator limits, thermal energy storage dynamics, and plant layout. Their resulting optimization problem is modeled as a mixed-integer linear program with logical and continuous variables. A method for MPC, which incorporates the variance of the outdoor temperature, is presented by Cadau et al. [22] for a large municipal building not connected to the DHN. In their approach, they use a dynamic model and MPC in Matlab/Simulink to control the internal building temperature by using dynamic programming. From the dynamic model a simplified grey-box model is incorporated into their MPC, where the cost function to minimized is the overall energy consumption of the building. Giraud et al. [23] use a load prediction model as input in their MPC approach to incorporate production planning and to minimize the supply temperature. By using a combination of linear constraints on different aspects of a DHN and a linearized model of the DHN a Mixed Integer Linear Program is derived.

A running theme amongst the presented control approaches is the reduction of network supply and return temperatures. The benefits associated with reducing the supply temperature have been shown to reduce distribution heat losses [24], and increase energy efficiency [25]. Additionally, operating at lower supply temperatures can reduce the degree of thermal stress, reducing potential leakages and maintenance [19]. In regards to the return temperature, lower return temperatures improve CHP efficiency by making it possible to extract more heat during flue gas condensing.

### 2.3. Motivation and Contributions

Through the development and validation of a dynamic model it is feasible to assess the influence that varying network inputs have on the systems performance, i.e., DHN heat losses and temperature propagation. The multitude of control approaches are numerous, but when it pertains to dealing with network delays and the associated propagation of heat, energy savings can be achieved with better management. Feed-Forward Model Predictive Control (FFMPC) can be implemented to reduce the supply and return temperatures in a DHN while maintaining a more consistent return temperature and upholding the thermal requirements of the end-user. The identification of a predictive model to be used within an MPC is not straight forward and the transparency in the literature in regards to this as well as the horizons used is limited. The contributions of this work is to address the following two questions:

1. What are the potential energy savings from implementing feed-forward model predictive control in district heating networks?
2. To what level of certainty is required, in regards to feed-forward signals, in order to reduce the supply and return temperatures in a district heating network?

## 3. Method

In this section, a short description of the DHN is first presented in order to highlight the motivation of this work. Thereafter, the physical model development, model equations and structure are explained. The system of equations used to predict heat propagation and heat delays are important for accurately modelling distribution temperatures over any distances within a DHN. This is proceeded by introducing model predictive control.

### 3.1. Description of the District Heating Network

**Figure 1:**
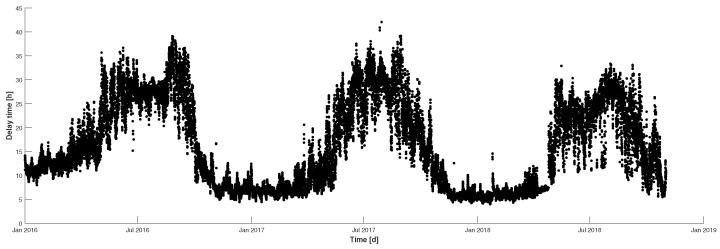
Seasonal variation in delay time for heat being supplied to Tillberga.

The DHN presented in this study is an aggregated perspective of the region Tillberga, which is situated in Västerås, Sweden, approximately 14.5 km from the heat supplier. The reasons for choosing Tillberga are due to the fact of having access to historical network measurements, and because it largely consists of residential housing, where as of 2018, was recorded having a population of 2164 inhabitants [26]. By analysing the historical network measurements from 2016 to 2018 it was determined that the heat demand ranged from 400 kW in the summer to 7.5 MW in the winter. During this period it was also observed that the highest and lowest outdoor temperatures measured were 32 ${}^{\circ }C$ and −21 ${}^{\circ }C$ respectively, and are inversely related to the heat demand. Therefore, as the outdoor temperature drops, the requirements for heating will also increase, but the delay of the heat from supply to consumption is in continuous flux. This is due to the fact that the delay time is a function of the network mass flow rate, pipe diameter, and length. Since pipe dimensions are fixed in established networks, the delay time will be arbitrated by the network mass flow. Illustrated in Figure 1 is a depiction of the delay time for Tillberga from 2016 to 2018. It can be observed that the seasonal variation plays a large roll in the delay time. During the winter periods, i.e., November to March, when the outdoor temperatures are typically below 0 ${}^{\circ }C$, the delay times are at the lowest points in Figure 1. This is a result of continuous heating requirements by the end-users, which facilitates a higher network mass flow, and therefore lower delay times, where the opposite effect happens during the warmer periods of the year. From the analysis of the variation in distribution delays it can be observed that the shortest historical delay achieved during this time period is approximately 5 h. Therefore, the injection of heat from the supplier needs to be initiated at least 5 h before it is required by the end-user.

### 3.2. Physical Model

The model presented has been developed in Dymola, version 2018, by Dassault Systéms. Dymola offers a simulation environment that is capable of handling complex dynamic systems by solving ordinary and algebraic differential equations. The programming language Modelica is used to design individual components to represent a DHN, i.e., pipes, valves, pumps, junctions, and heat consumers, in a physical way.

**Figure 2:**
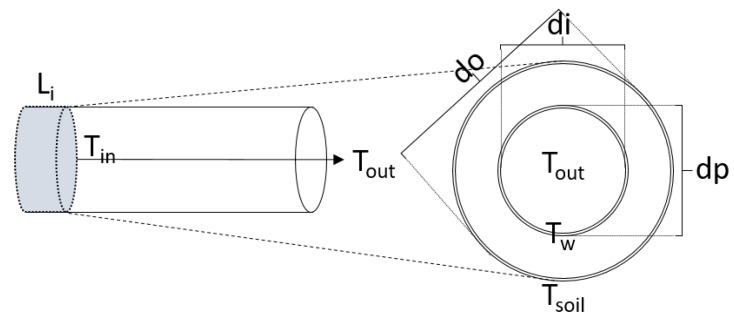
Principle components of the pipe model.

The goal in a DHN is to be able to supply the end-user with enough hot water, i.e., heat, to match their needs. Traditionally, this is accomplished on the heat generation side by injecting higher than necessary temperatures into the grid in order to meet customer demands. Yliniemi [27] reports that for the case of Sweden, which produces about 50 TWh, a potential savings of 50 million Swedish Kronor can be achieved by reducing the network distribution temperature by one degree. Therefore, when modelling a DHN it is essential to accurately predict the network temperature propagation, and is of significant interest in regards to the customers located the farthest distance away from the CHP. In order to capture the temperature dynamics as the heat propagates through the network a pipe model has been developed. Illustrated in Figure 2 are the principle components of the pipe that are incorporated into the model. The shaded area represents a given control volume length $L_{i}$ for a pipe of any length. The temperature of the water exiting the pipe, and therefore the ability to calculate heat propagation, is calculated using Equation (1):

(1) $$\begin{array}{c} \frac{dT_{\mathrm{out},i}}{dt}=\frac{\dot{m}c\left(T_{\mathrm{in},i}-T_{\mathrm{out},i}\right)-{\dot{Q}}_{loss}}{M_{i}c} \end{array}$$

where $T_{\mathrm{out},i}$ ( K) is the calculated temperature out of control volume *i*, $\dot{m}$ ( kg/$s^{-1}$) is the mass flow rate, *c* ( kJ ${kg}^{-1}$ $K^{-1}$) is the specific heat of the working medium, $T_{\mathrm{in},i}$ ( K) is the temperature of the water entering the control volume *i*, and ${\dot{Q}}_{loss}$ ( kW) is the calculated rate of heat loss defined by Equation (2):

(2) $$\begin{array}{c} {\dot{Q}}_{loss}=UA_{\mathrm{pipe}}\left(T_{w,i}-T_{\mathrm{soil}}\right) \end{array}$$

where *U* ( kW $m^{-2}$ $K^{-1}$) is the heat transfer coefficient, *A* ( m${}^{2}$) is the cross-section area of the pipe, $T_{w}$ ( K) is the temperature of the pipe wall, $T_{\mathrm{soil}}$ ( K) is the temperature of the ground surrounding the pipe, and $M_{i}$ is the mass of the inventory and can be calculated for each control volume as $M_{i}=A_{pipe,i}\;\cdot \;\rho \;\cdot \;L_{i}$ (kg). The heat transfer coefficient was calculated from Equation (3):

(3) $$\begin{array}{c} U={\left(\frac{d_{i}\;\cdot \;ln\left(\frac{d_{p}}{d_{i}}\right)}{2\lambda_{p}}+\frac{d_{i}\;\cdot \;ln\left(\frac{d_{o}}{d_{p}}\right)}{2\lambda_{i}}\right)}^{-1} \end{array}$$

where $d_{i}$, $d_{p}$, and $d_{o}$ (m) are the pipe diameters referenced in Figure 2 for the internal pipe inside diameter, internal pipe outside diameter, and the outer most diameter of the pipe respectively. $\lambda_{p}$ and $\lambda_{i}$ are the conductive heat transfer coefficients ( kW $m^{-2}$ $K^{-1}$) for water and the polyurethane insulation around the pipe. The temperature of the pipe wall $T_{w}$ ( K) was calculated from Equation (4):

(4) $$\begin{array}{c} \alpha \pi d_{i}L_{i}\left(T_{\mathrm{out}}-T_{w}\right)+U\pi d_{i}L_{i}\left(T_{\mathrm{soil}}-T_{w}\right)=\rho_{p}\frac{\pi (d_{p}^{2}-d_{i}^{2})}{4}cL_{i}\frac{dT_{w}}{dt} \end{array}$$

where α is the convective heat transfer coefficient ( kW $m^{-2}$ $K^{-1}$) of water, $L_{i}$ (m) is the length of the control volume, $\rho_{p}$ is the pipe wall density ( kg $m^{-3}$), and *c* is the specific heat of the pipe wall ( kJ ${kg}^{-1}$ $K^{-1}$). The convective heat transfer coefficient α was calculated from Equation (5):

(5) $$\begin{array}{c} \alpha =\frac{Nu\lambda_{w}}{d_{i}} \end{array}$$

where Nu is Nusselt’s number and was calculated from Equation (6):

(6) $$\begin{array}{c} Nu=\left[\frac{f}{8}\left(Re-1000\right)Pr\right]\;\cdot \;{\left[1+12.7{\left(\frac{f}{8}\right)}^{0.5}\left(Pr^{\frac{2}{3}}-1\right)\right]}^{-1} \end{array}$$

where *f* is the friction factor, which can handle laminar and turbulent flows, was calculated from Equations (7) and (8) by using the Reynold’s number Equation (9):

(7) $$\begin{array}{c} f=8{\left[{\left(\frac{8}{Re}\right)}^{12}+\frac{1}{{\left(\omega_{1}+\omega_{2}\right)}^{1.5}}\right]}^{\frac{1}{12}} \end{array}$$

(8) $$\left\{ \begin{array}{c} \omega_{1}={\left[-2.457\;\cdot \;\ln\left({\frac{7}{Re}}^{0.9}+0.27\frac{\epsilon }{d_{i}}\right)\right]}^{16}\\ \omega_{2}={\left(\frac{37530}{Re}\right)}^{16} \end{array}\right.$$

(9) $$\begin{array}{c} Re=\frac{\rho_{w}vd_{i}}{\mu } \end{array}$$

where $\rho_{w}$ ( kg $m^{-3}$) is the density of water, *v* ( ${ms}^{-1}$) is the velocity calculated from the mass flow rate, $d_{i}$ is the pipe inside diameter in meters, and μ ( Ns $m^{-2}$) is the water viscosity. From the heat losses described in Equation (2) it is also possible to estimate the temperature loss over a defined control volume, and has been calculated by using Equation (10):

(10) $$\begin{array}{c} T_{loss}=\frac{{\dot{Q}}_{loss}}{\dot{m}c} \end{array}$$

where $T_{loss}$ ( K) is the temperature loss, $\dot{m}$ ( kg $s^{-1}$) is the mass flow rate, *c* ( kJ ${kg}^{-1}$ $k^{-1}$) is the specific heat, and ${\dot{Q}}_{loss}$ was calculated from Equation (2). In order to determine the temperature propagating through the network or the new temperature after heat is consumed or lost, Equation (1) can be modified into Equation (11):

(11) $$\begin{array}{c} \frac{dT_{\mathrm{out}}}{dt}=\frac{\dot{m}c\left(T_{\mathrm{in}}-T_{\mathrm{out}}\right)-{\dot{Q}}_{loss}-{\dot{Q}}_{\mathrm{load}}}{Mc} \end{array}$$

where the addition of ${\dot{Q}}_{\mathrm{load}}$ represents the rate of heat (kW) consumed by the end user. It should be noted that for all calculations, since water is incompressible, it is assumed that the density, specific heat, and viscosity remain constant. If ${\dot{Q}}_{\mathrm{load}}$ is unknown, but the mass flow rate, inlet and outlet temperatures are known it is possible to calculate the rate of heat being consumed, by utilizing Equation (12):

(12) $$\begin{array}{c} {\dot{Q}}_{load}=\dot{m}c\left(T_{in}-T_{out}\right) \end{array}$$

With an accurate representation of heat as it moves through a given pipe, it is also of interest to determine how long, delay time τ, it takes for the temperature being supplied from the CHP to reach any given location downstream. This is achievable by knowing the installed pipe area, length, and mass flow rate of the water, which is represented in Equation (13):

(13) $$\begin{array}{c} \tau =\frac{A_{pipe}\rho L}{\dot{m}} \end{array}$$

where $A_{pipe}$ ( m${}^{2}$) is the inside pipe cross-sectional area and L ( m) is the length of the pipe. The density ρ is taken to be constant at 990 kg $m^{-3}$ and $\dot{m}$ is the mass flow rate of the distribution line. In order to estimate the amount of heat in the distribution and return lines over a specified time period, and the rate at which heat is being transferred (heat flux), Equations (14) and (15) were respectively calculated:

(14) $$\begin{array}{c} Q=\int \dot{m}c\left(T-T_{ref}\right)\;dt \end{array}$$

(15) $$\begin{array}{c} \dot{Q}=\dot{m}c\left(T-T_{ref}\right) \end{array}$$

where *Q* ( kJ) is the amount of heat over a specified time period for either the network’s supply or return line, $\dot{m}$ ( kg $s^{-1}$) is the mass flow rate, *c* ( kJ ${kg}^{-1}$ $K^{-1}$) is the specific heat of the water, *T* ( K) can either be the CHP supply temperature or the end-user return temperature, $T_{ref}$ ( K) is the reference temperature, and $\dot{Q}\left(kW\right)$ is the intensity of the heat flow in either the supply or return line. The reference temperature in this case is considered to be 21 ${}^{\circ }C$ and represents the desired indoor temperature of the end-user. This reference temperature has been chosen on the premise of it being the temperature of the heat sink, and therefore no positive heat transfer is achievable below this temperature. Equations (14) and (15) are intended to establish a reference point so that the control results to be presented can be compared with historical values. Equation (14) will provide a quantitative perspective as to how much heat has been transported in the distribution and return lines over a specified time period and Equation (15) will provide insight into the rate at which this heat is flowing. The formulation of these two equations makes it possible to calculate the potential energy savings and to visualize the possibility of peak heat load reduction over time.

### 3.3. Model Predictive Control

MPC is a multivariate control method that utilizes an internal prediction model in order to predict the future behavior of a system over a determined prediction horizon while considering constraints. The model that the MPC uses in this work is a multiple-input-single-output (MISO) discrete-time ARMAX model (Auto-Regressive Moving Average model with eXogenenous inputs) that was estimated through system identification. The non-linear relationship between the inputs and output can be approximated from a linear model with fixed-time delays. When the MPC receives signals for the measured disturbances and feed-back, a sequence of control actions are calculated and an optimization algorithm minimizes a determined cost function in order to predict the future behavior [28].

### 3.4. Overview of Prediction Model

In order to estimate the discrete-time ARMAX model it was first important to choose an appropriate sampling time. Due to the long delays in the DHN from supply to consumption a sampling time of one hour has been used. The next step was to artificially generate, within operating limits, the necessary input signals into the physical model in order to generate an output response for the outputs of interest. In this work the input signals are: CHP supply temperature, mass flow rate, and end-user demand. Therefore, from the generated inputs and associated output it was possible to identify which signals are to be controllable (CHP supply temperature and mass flow rate), which signals are going to be a measured disturbance (end-user demand), and what is to be controlled (return temperature from the end-user). Different model orders and methods need to be tested in order to obtain an optimal fit. As a first choice, a simple first order model was derived by using the input-output data from the open loop simulation of the validated physical model, and can be seen in Equation (16):

(16) $$\begin{array}{c} y\left(k\right)=ay\left(k-1\right)+b_{1}u_{1}\left(k-\tau \right)+b_{2}u_{2}\left(k\right)+b_{3}u_{3}\left(k-\tau \right) \end{array}$$

where *y* is the return temperature, $u_{1-3}$ are the CHP supply temperature, mass flow rate, and end-user demand respectively, the parameters *a* and $b_{1-3}$ are the estimated parameters for return temperature, CHP supply temperature, mass flow rate, and end-user demand respectively. At time *k* it is possible to calculate the return temperature based on the previous return temperature, the current mass flow rate, and the heat demand. A fixed variable time delay τ is used to delay the supply temperature in order to estimate the actual temperature being received in the DHN.

This first order model was initially utilized as the MPC’s internal prediction model. A feed-forward model predictive controller (FFMPC) was then designed and developed in Matlab-Simulink by implementing a fixed six hour input-delay on the end-user heat demand where the physical model was connected to the MPC by using the Dymola-Simulink interface. The MPC block has three inputs: return temperature from the end-user, return temperature set-point, and measured disturbance, and two outputs: the control signals for CHP supply temperature and mass flow rate. The controller was then used to generate data in a closed loop simulation by making step-changes in the return temperature and the heat demand. The step-changes were generated by calculating the mean and standard deviation from historical process data, and a normal random distribution function was developed in order to generate the necessary steps in time. In this way, a good level of excitation can be achieved in both the amplitude and frequency domains, where the controller then has to a adjust the supply temperature and mass flow in order to maintain the return temperature set-point. This new set of input-output data was then used to identify a first order discrete-time ARMAX model, as defined by Equation (17):

(17) $$\begin{array}{c} A\left(z\right)y\left(k\right)=\sum_{i=1}^{m}B_{i}\left(z\right)u_{i}\left(k-nk\right)+C\left(z\right)e\left(k\right) \end{array}$$

where y(k) is the system output at time *k*, $u_{i}\left(k\right)$ are the system inputs, nk denotes any corresponding delay to the inputs, e(k) is the system disturbance, and due to there being three input signals m=3. A(z), $B_{i}\left(z\right)$, and C(z) are the polynomials defined by Equations (18)–(20), of degree na, nb, and nc respectively, where the polynomials are represented by the backward shift operator $z^{-1}$, i.e., $z^{-1}x\left(k\right)=x\left(k-1\right)$.

(18) $$\begin{array}{c} A\left(z\right)=1+a_{1}z^{-1}+...+a_{na}z^{-na} \end{array}$$

(19) $$\begin{array}{c} B_{i}\left(z\right)=b_{1}+b_{2}z^{-1}+...+b_{nb}z^{-nb+1} \end{array}$$

(20) $$\begin{array}{c} C\left(z\right)=1+c_{1}z^{-1}+...+c_{nc}z^{-nc} \end{array}$$

Various polynomial orders were selected and tested for na, nb, and nc. It was determined that na = 1, nb = [1 1 1], nc = 2, and nk = [6 1 6] (supply temperature, mass flow rate, and heat demand) provided a fit to the estimation data of 89.7% using prediction as the focus. A second set of data was randomly generated, as described above, in order to evaluate the model, and it was found that the ARMAX model provided a fit of 71%.

**Figure 3:**
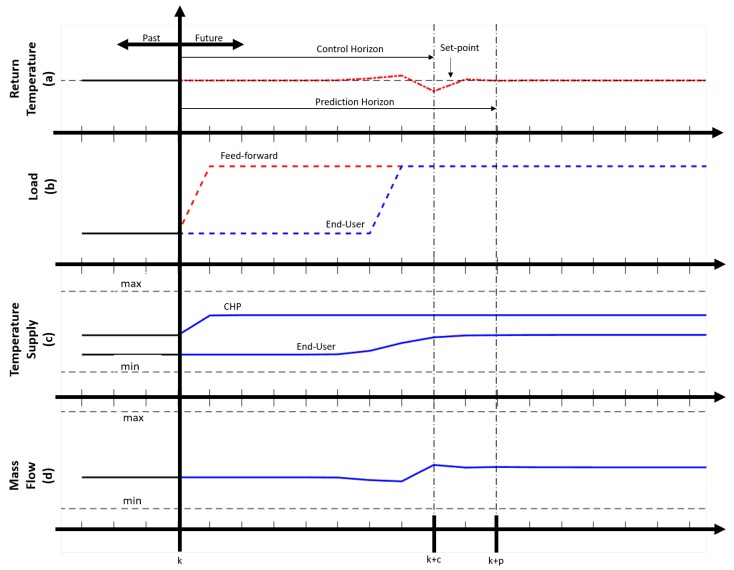
MPC step response: (**a**) return temperature operating around set-point, (**b**) feed-forward load and actual end-user load (**c**) actuation of CHP supply temperature and resulting end-user supply temperature, (**d**) actuation of mass flow rate.

The MPC’s performance when a step is initialized in the measured disturbance, i.e., end-user load, is illustrated in Figure 3. The feed-forward perspective of the controller can be observed in Figure 3b (red dash-line), at time *k* the MPC receives the load prediction 6 h in advance. Since the controller has the ability to receive information on the end-user load in advance it can increase the supply temperature from the CHP in order to accommodate the future influx of required heat, and is illustrated in Figure 3c. It can also be observed in Figure 3c that the supply temperature to the end-user is delayed due to the constant mass flow rate in Figure 3d. However, the step response shows that the supply temperature to the end-user begins to increase before the actual load is initialized at time k=6. The controller then takes the action of mitigating this temperature increase by adjusting the mass flow rate, as illustrated in Figure 3d, in order to reduce the amount of heat being supplied. Once the full extent of the load is observed, Figure 3b (blue dash-line), the controller produces an increase in the mass flow rate. This is why there is an observable decrease in the return temperature in Figure 3a. However, as the full extent of the supply temperature has not yet reached its final value, the controller makes a series of small adjustment to the mass flow where it can then be observed that the return temperature is able to settle around its set-point. Within the MPC, other parameters of interest, which require tuning are the prediction and control horizons.

Given the current control interval *k* and any observed measured disturbances, the prediction horizon *p* is the required number of future control intervals that the MPC needs to evaluate when determining the manipulable variables. Since the prediction horizon is discrete at each control step, the determined controller outputs are only observed at each time-step. The prediction horizon then moves forward one step and reevaluates and updates the system variables. In this case, the manipulated variables are the CHP supply temperature and mass flow rate. The control horizon *c* is the number of manipulable variable moves to be optimized at the control interval *k*, where at each control interval they are updated and reevaluated. For this MPC, the prediction and control horizons used are 8 and 10 respectively. This is because the mass flow rate has faster dynamics than the supply temperature, meaning that an instantaneous change in the flow rate is observable in the return temperature, as illustrated in Figure 3a,d. It was determined that using the mentioned horizons allows for the supply temperature to change, due to the delay, and that the mass flow rate reacts when there is a substantial change in the return temperature.

## 4. Results and Discussion

### 4.1. Validation

**Figure 4:**
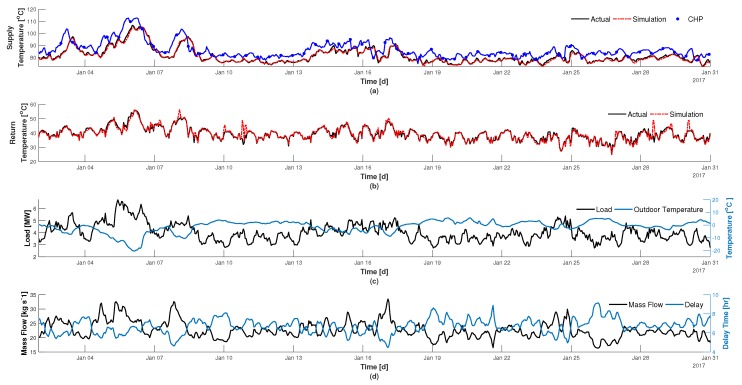
Validation of DHN model: (**a**) temperature supplied to Tillberga in comparison to the temperature being supplied from the CHP, (**b**) return temperature to CHP from Tillberga, (**c**) load and outdoor temperature, (**d**) mass flow rate and delay time.

Illustrated in Figure 4 are the achieved results for simulation the supply and return temperatures for Tillberga for January 2017. The only inputs utilized in the model are historical values for the temperature and mass flow rate being supplied from the CHP, the ground temperature, and the load. It can be observed in Figure 4a that the model can adequately predict the heat propagating, and losses through the network over a distance of 14.5 km, i.e., the simulated (red dash-line) are following the trends of the actual temperature (black line). It is also possible to take note of the distribution delay and losses since there is a noticeable shift in the temperature being supplied from the CHP (blue-dot line) and a 2–5 ${}^{\circ }C$ drop in the supplied temperature. The modelled vs. actual return temperature in Figure 4b is also within good agreement, and is calculated using the load from Figure 4c by using Equation (12), and mass flow rate in Figure 4d. The mass flow rate in Figure 4d is coupled with the delay time in distribution temperature, i.e., the amount of time it takes for the temperature leaving the CHP to arrive to Tillberga. During this period, the maximum and minimum observed flow rates were 33.5 and 14 kg $s^{-1}$ with a corresponding delay of 4.4 and 10.4 h respectively.

### 4.2. Control Implementation

The purpose of the presented control approach is to reduce DHN supply loads by lowering distribution temperatures while maintaining a more consistent (lower variance) and lower return temperature. This is accomplished by controlling the return temperature close to a set-point by using network supply temperature and mass flow rate as actuators, and a prediction of the load is used as a feed-forward signal to the controller. Substations in DHN control the amount of water in order to meet the variations in load demand which affects the pressure drop in that part of the network. The change in the pressure drop is sequentially resolved by circulation pumps since the pressure drop and water speed are directly linked. Furthermore, as the water flow increases so does the pressure drop since the water speed is proportional to the square root of the pressure difference [29]. Therefore, based on the previously mentioned connection, the mass flow in DHN can be viewed as a controllable action connected to circulation pumps. In the control results to be presented, it can be observed that the new control results remain within the limits of the historical mass flow rates, which implies that the pressure drop also remains within the network limits.

**Figure 5:**
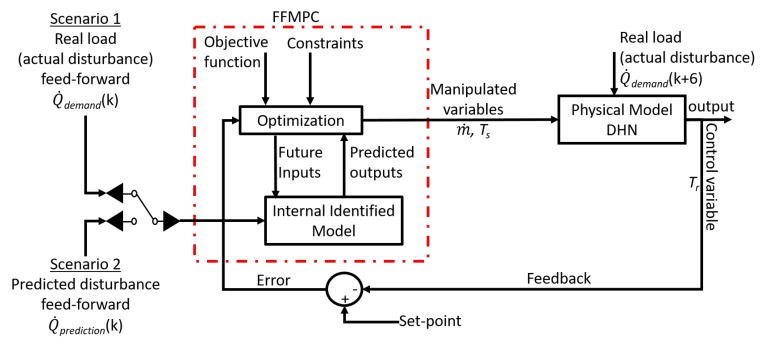
Overview of FFMPC implementation and feed-forward signal: Scenario 1—historical load and Scenario 2—prediction model.

By coupling the designed FFMPC with the validated physical model, it was possible to evaluate the new control performance against historical processes data as illustrated in Figure 5. The overview expresses a need for two explicit inputs: the actual end-user load (${\dot{Q}}_{demand}$ [MW]) at time k+6, and the actual load (${\dot{Q}}_{demand}$ [MW]) at time *k* is used as a measurable disturbance to the MPC.

The output signals from the FFMPC are the mass flow rate ($\dot{m}$) and supply temperature from the CHP ($T_{s}$). These signals are used as input into the physical model, which then calculates the network heat propagation as was illustrated in Figure 4a. At time k+6 the actual heat demand is injected into the physical model in order to calculate the return temperature to the CHP ($T_{r}$) which is the control signal of interest. The error that arises between the resulting return temperature and the desired temperature is assessed in the MPC. At time *k* the MPC utilizes the internal model and feed-forward signal to predict the trajectory of future inputs and output, where it attempts to minimize the error over the prediction horizon. The MPC only applies the first step in the optimal sequence to the physical model in order to achieve the desired return temperature. At time k+1 the controller obtains a new error, the prediction horizon shifts forward, and the process of obtaining optimal control moves repeats. Figure 5 outlines two scenarios: Scenario 1 simulates the case of knowing the exact level at which the disturbance will be initiated, i.e., feed-forward the real (historical) load, and Scenario 2 is evaluated by using a load prediction model. The prediction model was derived from historical load and ambient temperatures and is expressed in Equation (21):

(21) $$\begin{array}{c} {\dot{Q}}_{prediction}={\dot{Q}}_{k}+\left({\dot{Q}}_{avg_{k}}-{\dot{Q}}_{avg_{k+6}}\right)+c\left(T_{amb_{k+6}}-T_{amb_{k}}\right) \end{array}$$

where ${\dot{Q}}_{k}$ is the current load of the end-user [MW], ${\dot{Q}}_{avg}$ is the daily hourly average of the historical load for winter periods at the current time *k* and 6 h ahead k+6, $T_{amb}$ is the outdoor temperature at the current time *k* and six hours ahead k+6, and c signifies the impact of the outdoor temperature on the prediction.

**Figure 6:**
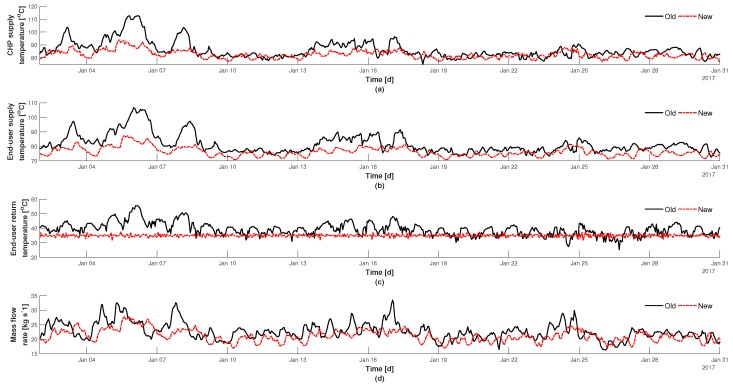
Implementation of FFMPC results and comparison between actual and controlled signals, Scenario 1. (**a**) supply temperature from CHP, (**b**) supply temperature to the end-user, (**c**) return temperature to CHP from the end-user, and (**d**) mass flow rate.

Illustrated in Figure 6 are the control results of Scenario 1 from implementing FFMPC for the month of January in 2017 using the load depicted in Figure 4c. The old (historical) and new control trends are represented as black- and red-plots respectively. It can be observed in Figure 6a that the new control scheme produces a lower CHP supply temperature. Therefore, a lower supply temperature and mass flow rate to the end-user can be achieved, as illustrated in Figure 6b,d respectively. The return temperature from the end-user has a set-point of 35 ${}^{\circ }C$ and it can be observed in Figure 6c that the controller is able to maintain the return temperature around this point with a much smaller variance compared to the old control strategy.

**Figure 7:**
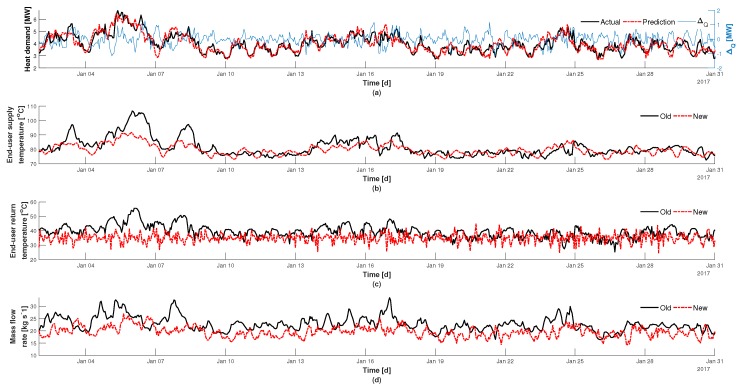
Control results for Scenario 2, using load prediction. (**a**) predicted load in relation to the actual load, (**b**) supply temperature to the end-user, (**c**) temperature leaving the end-user, and (**d**) mass flow rate of heat being delivered.

Illustrated in Figure 7 are the control results for Scenario 2. In this case the feed-forward load signal to the MPC was calculated from the prediction model. The assumption is that the controller will be acting on information that is currently available to the plant production management team. From the basis of ambient temperature prognosis it is possible to estimate the thermal needs of the end-user. It can be observed in Figure 7a that the load prediction model is able to follow the amplitude of the actual load request from the end-user quite well, where the difference is represented by $\Delta_{Q}$. It can be noticed in Figure 7c that the controller has a difficult time managing the desired set-point of 35 ${}^{\circ }C$. This can be attributed to the fact that the feed-forward signal is only a prediction of the actual heating needs. Therefore, the controller’s supply temperature to the end-user illustrated in Figure 7b is not an accurate representation of peak load requirements. Based upon this miss-match it can be observed in Figure 7d that the mass flow rate is adjusted, but is unable to effectively accommodate for deviations in the predicted load in regards to the actual load. The average and standard deviation for the new controlled return temperature are found to be μ=35 and σ=3.1 respectively, where the standard deviation is roughly three times higher than it was for Scenario 1. This highlights the importance of the feed-forward signal. A better prediction model would lead to a lower variance in the return temperature, as is illustrated in Figure 6c, which suggest the potential for reducing the return temperature set-point and further increasing energy savings. From the normal distribution it is then determined that for 88.5% of the time the return temperature is within the bounds of ± 5 ${}^{\circ }C$ of the set-point. For the other 11.5% of the time the maximum and minimum new return temperatures are found to be 24.3 ${}^{\circ }C$ and 44.8 ${}^{\circ }C$ respectively, which is still within the limits of the old control in Figure 7c.

### 4.3. Control Performance and Assessment

**Figure 8:**
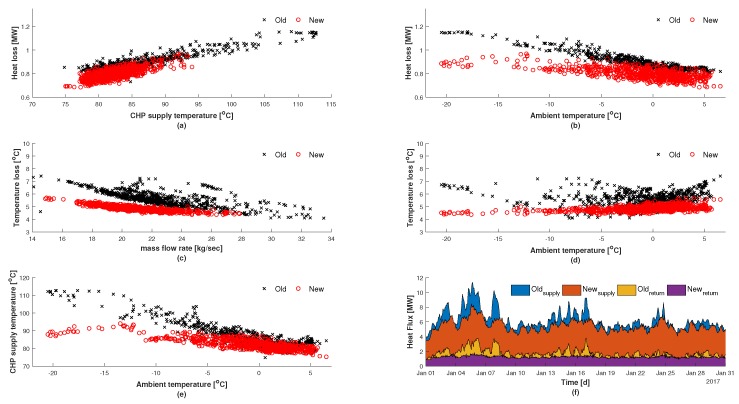
Improvements gained from Scenario 1 (feed-forward historic load). (**a**,**b**) represent the associated heat losses in respects to CHP supply temperature and the ambient temperature respectively; (**c**,**d**) represent the temperature losses in respects to the mass flow rate and ambient temperature respectively; (**e**) CHP supply temperature in respects to ambient temperature; (**f**) Comparison between historical values (old) and control values (new) in regards to rate of heat supplied and returned in the network.

An evaluation of the controller’s performance in regards to Scenario 1 has been assessed in order to highlight the potential improvements and is illustrated in Figure 8. From the control results obtained in Figure 6 it was shown that utilizing FFMPC led to a reduction not only in frequency but also in amplitude of the CHP supply temperature and the network mass flow rate. The added value of developing and validating a physical model of the system provides the means to compare historical values to the new control results, since the new approach allows for a lower distribution temperature. It can be observed in Figure 8a that the highest and lowest associated heat losses over the duration of the distribution line from a historical view point are 1.15 MW and 0.73 MW respectively, with an average overall heat loss of 0.89 MW. The heat loss calculation was calculated using Equation (2), where it was shown in Figure 4 that the system of equations used can predict the propagation of heat and associated losses. In comparison, the highest and lowest heat losses over the distribution line from Scenario 1 are 0.97 MW and 0.69 MW respectively, with an average overall heat loss of 0.80 MW. This results in an average reduction of 10.5% compared to the historical value. Illustrated in Figure 8b it can be observed that the heat losses are reduced despite the outdoor ambient temperature, which varies from −20 ${}^{\circ }C$ to 5 ${}^{\circ }C$. Illustrated in Figure 8c,d are the calculated temperature losses, using Equation (10), from the CHP to the end-user in respects to the mass flow rate and ambient outdoor temperature respectively. The average temperature loss for the historical and control perspectives are 5.6 ${}^{\circ }C$ and 4.9 ${}^{\circ }C$ respectively. In Figure 8c it can be observed that the historical values for mass flow rate exceed the controlled maximum value of 27.85 kg $s^{-1}$. This highlights the fact that even with overall lower network flow rates, it is still possible to reduce the network temperature losses. The reduction in losses can be further explained by the reduction in the network supply temperature as illustrated in Figure 8e. The heat flux in the supply and return lines is illustrated in Figure 8f, and was derived by using Equation (15). In regards to the heat being supplied to the end-user, the new control case shows the potential in peak load reduction, i.e., Old_supply_–New_supply_. The average reduction in peak load being supplied to the end-user is found to be 0.78 MW with a maximum reduction of 4.2 MW. For the case of the return heat, New_return_, the control results lead to more stability in the return line due to the reduced variance in the mass flow rate. From the supply and return profiles it was possible to calculate an average overall savings of 254 kW by using Equation (22):

(22) $$\begin{array}{c} \bar{X}=\frac{\sum_{i=1}^{n}\left[\dot{m}c{\left(T_{s_{i}}-T_{r_{i}}\right)}_{historic}-\dot{m}c{\left(T_{s_{i}}-T_{r_{i}}\right)}_{control}\right]}{n} \end{array}$$

where Ts and Tr are the CHP supply and end-user return temperatures ( K) for the historic (old) and control (new) results respectively, $\dot{m}$ ( kg $s^{-1}$) is the mass flow rate in the DHN and is considered to be the same in the supply and return lines, *c* is the specific heat ( kJ ${kg}^{-1}$K), *i* is the index of the summation starting in January, and *n* is the number of samples.

To quantify the performance of each control scenario a performance assessment is outlined in Table 1. For each scenario the supply and return streams are outlined accordingly, where the historical values are the same for each case. To assess the performance of the two scenarios the average and standard deviations are taken for the supply-, return temperature, and the mass flow rate. The performance of Scenario 1 shows that the controller is able to supply a lower average temperature of 82.3 ${}^{\circ }C$.

It was illustrated in Figure 8a that a reduction in the CHP supply temperature leads to lower losses, where it is calculated that a heat reduction of 12.5% can be achieved with the new supply temperature. The reduction in the heat supplied was calculated by taking the percent difference of the heat calculated in Table 1, which is based on Equation (14). In regards to the mass flow rate the performance of Scenario 1 is able to reduce the average mass flow rate from 22.6 kg $s^{-1}$ to 21 kg $s^{-1}$. The calculated standard deviation of the new mass flow rate also shows that there is less variation in the network flow which reduces the possible stress on the network. The performance of Scenario 2 shows a heat reduction of 13.7% on the supply side, the highest of both cases. The average supply temperature is found to be higher than Scenario 1 and is why the average mass flow rate is lower than Scenario 1. This is because a lower mass flow rate means that there is more time for heat to be extracted from the network and therefore a higher temperature is required. The prediction model used, illustrated in Figure 7a, shows that the predicted load (feed-forward disturbance) follows the amplitude of the historical values quite well. The difference between the historical and predicted load illustrates a fluctuation of approximately ± 1 MW, and explains why the variation around the return temperature set-point exhibits a high degree in excitation.

**Figure 9:**
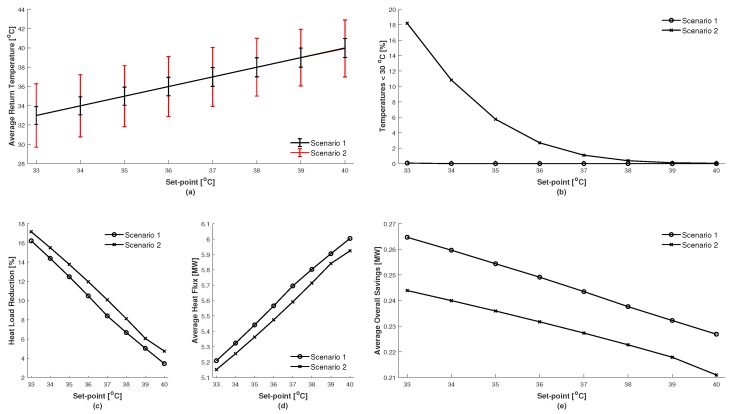
Sensitivity test from varying the set-point on the return temperature for each scenario: (**a**) average return temperature, (**b**) percentage of temperatures occurring below 30 ${}^{\circ }C$, (**c**) potential percentage in heat load reduction, (**d**) average rate of heat supplied, and (**e**) average overall savings potential.

In regards to the contingency of the controller’s capability of managing a proposed set-point of 35 ${}^{\circ }C$, a sensitivity analysis for varying set-points is illustrated in Figure 9. In Figure 9a the average return temperatures for each scenario are given in respects to the change in set-point. The error bars represent 1 standard deviation and therefore 68% of the return temperature values can be considered to be within these regions. Illustrated in Figure 9b are the percentages of return temperatures found to be below 30 ${}^{\circ }C$. If it is considered that 30 ${}^{\circ }C$ is the lowest allowable limit for a return temperature, then Scenario 1 could have a return temperature set-point of 33 ${}^{\circ }C$, and Scenario 2 would require a set-point of 38 ${}^{\circ }C$. Figure 9c illustrates the potential in the heat load reduction from the CHP. It is interesting to note that Scenario 1 under-performs in comparison to Scenarios 2. However, this can be explained by observing Figure 9d, in which the average rate of heat being supplied for the control period is given at each set-point step. It displays that the average rate of heat supplied in Scenario 1 exceeds that of Scenario 2 and that the percentage of peak load reduction is reflected in this. This also suggests that the use of a load prediction can potentially underestimate the load requirements. Figure 9e displays the impact of being able to control the return temperature more consistently, and is calculated using Equation (22). Since Scenario 1 is able to maintain the set-point with the most consistency it can be observed that Scenario 1 provides the highest potential for savings because the set-point can be set much lower than Scenario 2.

## 5. Conclusions

Presented in this work is a method for the modelling and control of a district heating network. The prospects of evaluating the thermal dynamics inherent in district heating networks is achievable through the development of a simplified dynamic physical model. Furthermore, the validation of the physical model provides the basis from which model based control can be implemented. From the identification process it was determined that a first order discrete-time ARMAX model could be used as the model predictive controller’s internal model. In order to determine the feasibility of reducing network supply temperatures and to assess the potential energy savings two scenarios were evaluated. It was determined that it is achievable to reduce the average network supply temperature by 3.7 ${}^{\circ }C$ and 4 ${}^{\circ }C$ respectively, and the percent in CHP heat load reduction is found in each scenario to be 12.5%, and 13.7% respectively for the analysed time period.

Model predictive control with feed-forward shows a significant potential in reducing network return temperatures and peak load production, but is subject to the quality of the signal being fed-forward in time to the controller. This is illustrated by comparing Scenarios 1 and 2, where a prediction model approach could limit the return temperature set-point. If a lower limit of the return temperature is to be required, i.e., 30 ${}^{\circ }C$, then the set-point of the controller will be dictated by the quality of the feed-forward signal. This is apparent when looking at Scenario 2, which would require a return temperature set-point of 38 ${}^{\circ }C$ due to the degree of variance observed in the return temperature. However, despite this increase in the set-point, Scenario 2 would still be able to provide an average overall savings of 222 kW. Therefore, the inclusion of more accurate load prediction models would enable the ability to reduce the return temperature set-point due to a lower variation in the return temperature signal, and would contribute to higher energy savings. Faster sampling time could also be considered, since the feedback would then be able to compensate for poor load predictions earlier.

## Figures and Tables

**Table 1:** Performance comparison for the two observed cases.

	Historic		Scenario 1		Scenario 2	
	Supply	Return	Supply	Return	Supply	Return
Temp. avg. ( ${}^{\circ }C$)	86	39.2	82.3	35	86	35
Temp. std. ( ${}^{\circ }C$)	6.6	4.4	3.1	0.9	3.7	3.1
Temp. loss avg. ( ${}^{\circ }C$)	5.6	-	4.8	-	5.3	-
mass flow avg. ( kg $s^{-1}$)	22.6	-	21	-	19.4	-
mass flow std. ( kg $s^{-1}$)	3	-	2.2	-	2.2	-
Heat ( MJ)	16.65 × 10^6^	47 × 10^5^	14.57 × 10^6^	33.1 × 10^5^	14.36 × 10^6^	30.5 × 10^5^
Heat reduced (%)	-	-	12.5	29.6	13.7	35

avg. = average; std. = standard deviation; ’-’ = not applicable. Scenario 1: feed-forward of historical load, Scenario 2: feed-forward of prediction load.
